# Targeted *Ptpn11* deletion in mice reveals the essential role of SHP2 in osteoblast differentiation and skeletal homeostasis

**DOI:** 10.1038/s41413-020-00129-7

**Published:** 2021-01-27

**Authors:** Lijun Wang, Huiliang Yang, Jiahui Huang, Shaopeng Pei, Liyun Wang, Jian Q. Feng, Dian Jing, Hu Zhao, Henry M. Kronenberg, Douglas C. Moore, Wentian Yang

**Affiliations:** 1 Department of Orthopedic Surgery, Brown University Alpert Medical School and Rhode Island Hospital, Providence, RI 02903 USA; 2 Department of Mechanical Engineering, University of Delaware, Newark, DE DE19716 USA; 3 Department of Biomedical Sciences, Texas A&M College of Dentistry, 3302 Gaston Ave, Dallas, TX 75246 USA; 4 Department of Comprehensive Dentistry, Texas A&M College of Dentistry, 3302 Gaston Ave, Dallas, TX 75246 USA; 5 Endocrine Unit, Massachusetts General Hospital and Harvard Medical School, Boston, MA 02114 USA

**Keywords:** Bone, Pathogenesis

## Abstract

The maturation and function of osteoblasts (OBs) rely heavily on the reversible phosphorylation of signaling proteins. To date, most of the work in OBs has focused on phosphorylation by tyrosyl kinases, but little has been revealed about dephosphorylation by protein tyrosine phosphatases (PTPases). SHP2 (encoded by *PTPN11*) is a ubiquitously expressed PTPase. *PTPN11* mutations are associated with both bone and cartilage manifestations in patients with Noonan syndrome (NS) and metachondromatosis (MC), although the underlying mechanisms remain elusive. Here, we report that SHP2 deletion in bone gamma-carboxyglutamate protein-expressing (*Bglap*^+^) bone cells leads to massive osteopenia in both trabecular and cortical bones due to the failure of bone cell maturation and enhanced osteoclast activity, and its deletion in *Bglap*^+^ chondrocytes results in the onset of enchondroma and osteochondroma in aged mice with increased tubular bone length. Mechanistically, SHP2 was found to be required for osteoblastic differentiation by promoting RUNX2/OSTERIX signaling and for the suppression of osteoclastogenesis by inhibiting STAT3-mediated RANKL production by osteoblasts and osteocytes. These findings are likely to explain the compromised skeletal system in NS and MC patients and to inform the development of novel therapeutics to combat skeletal disorders.

## Introduction

Osteoblasts (OBs) are formed by mesenchymal stem cells (MSCs) originating from the neural ectoderm and paraxial and lateral plate mesoderm.^1^ Neural ectoderm-originating OBs are formed directly from condensed mesenchyme tissue without going through the intermediate stages. Such OBs generate most of the cranial squamous bones and the lateral part of the clavicle. The remainder of the skeleton is generated by OBs from the paraxial and lateral plate mesoderm and is first formed of an intermediate cartilaginous analog. The MSCs that form OBs also give rise to fat and cartilage cells. At the end of osteogenesis, mature OBs are embedded in the bone as osteocytes that regulate matrix mineralization and bone quality.

Osteogenesis is a dynamic process that involves multiple steps, such as lineage segregation of MSCs, osteogenic differentiation, and maturation.^2,3^ These osteogenic events rely heavily on the reversible phosphorylation of signaling proteins, including but not limited to SOX9 (ref. ^4,5^), RUNX2 (ref. ^6,7^), OSTERIX,^8,9^ β-CATENIN,^10,11^ and PPAR-γ.^12,13^ RUNX2 crucially modulates the osteogenic commitment of MSCs and the subsequent maturation and expression of extracellular matrix proteins.^6^ RUNX2 has a variety of targets, such as OSTERIX,^2^ type I collagen (COL1a1),^14^ and alkaline phosphatase (ALP).^15^ When immature OBs mature and become functional OBs, they begin to express high levels of osteocalcin, which is also known as bone gamma-carboxyglutamate protein (BGLAP). Finally, mature OBs are embedded in the bone matrix to become osteocytes that express high levels of dentin morphogenic protein 1 (DMP1)^16^ and sclerostin (SOST).^17^ DMP1 promotes bone matrix mineralization by binding Ca^2+^ because of its acidic nature,^18^ while sclerostin antagonizes Wnt signaling to inhibit bone formation by OBs.^19,20^ Aberrant phosphorylation of these signaling proteins during development and adulthood can impact OB development and function, matrix mineralization and bone quality.^2^ OB dysregulation is the cause of several metabolic, genetic, and oncogenic skeletal disorders,^21,22^ none of which has an effective medical treatment. To date, most of the work on protein phosphorylation in OBs has focused on protein tyrosine kinases^23^ and relatively little on protein tyrosine phosphatases, which, however, can have equally profound functional significance.

OBs are also crucial in regulating osteoclast differentiation and skeletal remodeling.^24^ Several soluble factors produced by OBs, e.g., MCP1 (ref. ^25^), osteoprotegerin (OPG, encoded by *Tnfrsf11b*),^26^ receptor activator of nuclear factor kappa-B ligand (RANKL, encoded by *Tnfsf11*)^27^ and Sema3A,^28^ contribute to the crosstalk between OBs and osteoclasts. The mechanism by which RANKL and OPG modulate osteoclast development and function has been studied in detail.^27^ OBs produce RANKL, which stimulates RANK on osteoclast precursors, resulting in osteoclast differentiation via the activation of downstream signaling pathways such as NF-κB,^29^ c-Fos,^30^ and NFATc1 (ref. ^31^). This process can be blocked by OPG by suppressing RANKL binding to RANK.^32,33^ The coupled effect of bone formation by OBs and bone resorption by osteoclasts supports bone mass and maintains mineral homeostasis throughout our lifespan. However, the molecular and cellular mechanisms through which OBs modulate osteoclasts and bone mineral homeostasis in certain diseases remain elusive.

SHP2, which is encoded by *PTPN11*, is a ubiquitously expressed PTPase that plays an essential role in the development and/or maintenance of multiple organs and tissues.^34^ Mutations in *PTPN11* are associated with human Noonan syndrome,^35,36^ Leopard syndrome,^37^ and metachondromatosis (MC),^38–41^ all of which have severe skeletal manifestations. SHP2 has been reported to modulate the crosstalk between a series of signaling pathways triggered by fibroblast growth factors,^42^ hedgehog,^39^ bone morphogenetic proteins,^43^ and Wnts.^44^ Dysregulation of one or more of these signaling pathways is known to spatiotemporally influence subsequent osteogenic programs, such as OB maturation and ultimately endochondral and intramembranous ossification, however, the underlying molecular and cellular mechanisms remain elusive. Previously, SHP2 was shown to modulate the fate of osteochondral progenitors.^44^ In this study, we generated and characterized OB-specific SHP2-deficient mice and their cellular derivatives. Our work has led to the discovery of the pivotal regulatory role of SHP2 in osteoblastic-cell lineage development and bone mineral homeostasis.

## Results

### Mice lacking SHP2 in the *Bglap*^+^ cell lineage are crippled, osteopenic, and grow osteochondromas and enchondromas

To investigate the role of SHP2 in mature OBs and osteocytes, mice carrying *Ptpn11*-floxed (*Ptpn11*^*fl*^) alleles^39^ were crossed with *Tg(Bglap-Cre)*^45^ and *Tg(Bglap-CreER)*^46^ mice to generate SHP2CTR^Bglap^, SHP2KO^Bglap^, SHP2CTR^Bglap/ER^, and SHP2KO^Bglap/ER^ mice as described previously (Fig. S1a, b). SHP2KO^Bglap^ and SHP2KO^Bglap/ER^ mice treated with tamoxifen (TM) lacked SHP2 in mature OBs and osteocytes, and SHP2CTR^Bglap^ and SHP2CTR^Bglap/ER^ mice treated with TM served as controls. To trace the fate of *Bglap*^+^ osteoblastic cells in vivo, *Tg(Rosa26*^*ZSG*^*) (R26*^*ZSG*^*)*^47^ mice were bred to both control and SHP2 mutants as a reporter of Cre expression in selected lines. As expected, in both the *Tg(Bglap-Cre)* and *Tg(Bglap-CreER)* mice, the R26^ZSG^ reporter was expressed primarily in cells within the bone cortex, endosteum, and trabeculae, which was consistent with the locations of mature OBs and osteocytes (Fig. S1c). Importantly, immunostaining revealed that the level of SHP2 in R26^ZSG+^ *(Bglap*^+^*)* OBs and osteocytes of the trabecular and cortical bone of the SHP2KO^Bglap^ mice was markedly reduced compared to that of the SHP2CTR^Bglap^ littermate controls (Fig. S1d), indicating that *Tg(Bglap-Cre)* and *Tg(Bglap-CreER)* mediated robust excision of the *Ptpn11*-floxed alleles.

**Fig. 1:**
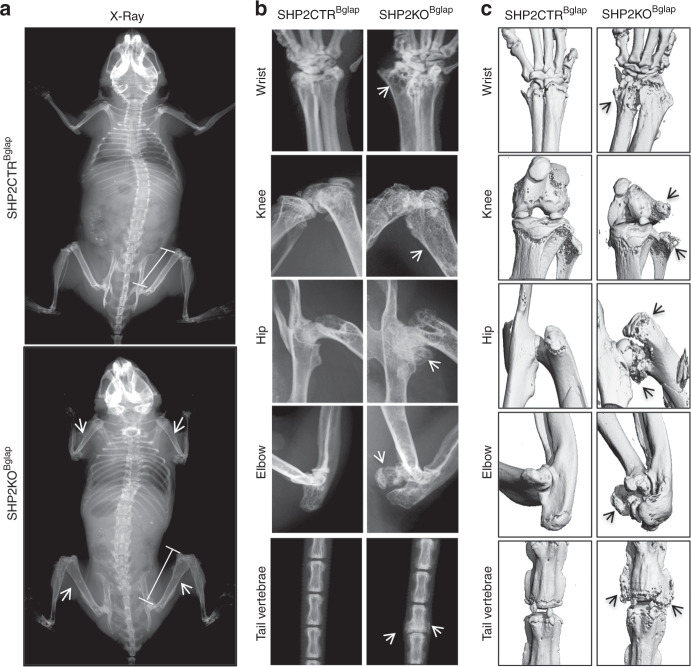
Mice lacking SHP2 in *Bglap*^+^ osteoblasts and osteocytes display skeletal dysplasia, reduced BMD, and the growth of enchondromas and osteochondromas. **a** Radiographs of 13-week-old SHP2CTR^Bglap^ and SHP2KO^Bglap^ mice showing the skeletal structure, femur length (lines), and BMD (arrows). X-ray (**b**) and µCT (**c**) radiographs of skeletal preps from the mice described in **a**. Note the reduced BMD, deformed knee and hip joints, and growth of osteochondromas in SHP2KO^Bglap^ mice (arrows) (*n* = 5)

SHP2KO^Bglap^ mice were born at the expected Mendelian ratios and were indistinguishable from the SHP2CTR^Bglap^ mice in terms of body weight and size up to 10 weeks of age (Fig. S2a). These observations are supported by the finding that ALP activity and the abundance of the osteogenic and chondrogenic marker genes *Runx2, Osx, Col1a1, Ibsp, Bglap, Col2a1*, and *Col10a1* transcripts were comparable in the newborn tibial frozen sections between the control and SHP2 mutants (Fig. S1e). However, the mobility of SHP2KO^Bglap^ mutants became progressively restricted as they aged (Fig. SV1). Analysis of skeletal morphology with high-resolution plain radiographs and microcomputed tomography revealed a striking skeletal phenotype that included scoliosis, reduced bone mineral density (BMD), growth of osteochondromas and enchondromas at or adjacent to the physis of the long bones and vertebrae, and an increase in the length of tubular bones (Figs 1a–c and S2a). Taken together, the morphological data suggest that normal SHP2 expression in *Bglap*^+^ osteoblastic cells is crucial for normal endochondral ossification, joint development and function, and bone mineral homeostasis.

### SHP2 deletion in *Bglap*^+^ osteoblastic cells compromises cancellous and cortical bone formation, matrix mineralization, and bone quality

**Fig. 2:**
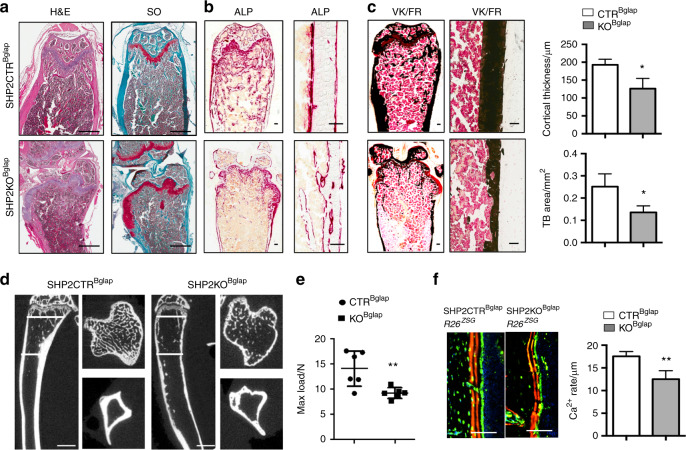
SHP2 deletion in *Bglap*^+^ cells leads to deformation of articular and growth plate cartilage, defective mineralization of cortical and cancellous bone, and bone volume reduction. **a** Distal femur paraffin sections stained with H&E and Safranin O (SO) demonstrate the structure and histology of articular and growth plate cartilage in 13-week-old mice with the indicated genotypes. Scale bar: 1 mm. Femoral frozen sections stained for ALP activity (**b**) and mineral deposition with the von Kossa (VK) method (**c**) of the mice described in **a**. The low-power full images are shown in Fig. S2c. Quantified cortical bone thickness and mineralized cancellous bone areas are presented as bar graphs on the right (*n* = 4, *P < 0.05, Student’s *t*-test). Scale bar: 100 μm. **d** Representative sagittal and transverse µCT radiographs show the microarchitecture of the proximal tibia from 10-week-old male mice with the indicated genotypes. The sectioning planes are marked by lines. Scale bar: 1 mm. **e** Scatter plots show the maximum load of the tibia from 10-week-old male mice determined using the 3-D bending test (*n* = 6, **P < 0.01, Student’s *t*-test). **f** Fluorescent images of femoral frozen sections showing new bone formation in 13-week-old mice that received xylenol orange injection at days 7 and 2 prior to euthanasia. Quantified data are shown by bar graphs on the right (*n* = 4, **P < 0.01, Student’s *t*-test). Scale bar: 100 μm

To investigate the skeletal pathology of SHP2KO^Bglap^ mice, we microscopically compared tibiae from 13-week-old control and knockout mice. Hematoxylin & eosin (H&E) and Safranin O-stained frozen sections demonstrated that the cartilaginous epiphysis was clearly separated from the diaphysis in the distal femur of SHP2CTR^Bglap^ mice by a well-organized growth plate. A large amount of trabecular bone formed in the metaphysis, which was stained strongly for ALP, and the trabecular bone and diaphyseal cortex were stained heavily with von Kossa (Fig. 2a–c; top). In contrast, these structures were disrupted in the SHP2KO^Bglap^ mutants, which exhibited expanded growth plate cartilage (Figs 2a, bottom and S1f), distorted and calcified articular cartilage of variable severity, and the growth of osteochondromas (Fig. 2a–c, bottom). The affected animals also had grossly reduced bone mass, cortical bone thickness, and ALP activity (Fig. 2a–c, bottom), accompanied by the massive accumulation of adipocytes in the bone marrow (Fig. S3a). The microscopic observations were corroborated by µCT-based histomorphometry analysis. The volumetric density (bone volume (BV)/total volume (TV)), connectivity density, and trabecular number were significantly reduced in the proximal tibiae of 10-week-old SHP2KO^Bglap^ mice compared to age- and sex-matched SHP2CTR^Bglap^ animals, while the structure model index, trabecular separation, and bone marrow cavity were markedly increased (Figs 2d and S2b, c). Three-point bending tests revealed that the maximum load to failure of 10-week-old SHP2KO^Bglap^ mutant mice was significantly reduced by 34.57%, compared to that of SHP2CTR^Bglap^ controls (Fig. 2e). Next, we examined the influence of SHP2 on the regulation of bone formation using dual xylenol orange labeling of mineral apposition in 13-week-old animals. The new bone formation rate, as expected, was lower in the SHP2 mutants than in the controls (by 16.79%, *P* < 0.01, Fig. 2f). Finally, to determine whether the defects in bone formation and matrix mineralization in the SHP2 mutants were due to impaired cell proliferation and/or viability, we carried out EdU labeling and immunostaining assays and found that the numbers of EdU- and cleaved-Caspase-3-positive cells were comparable in the frozen tibial sections of SHP2KO^Bglap^;R26^ZSG^ and SHP2CTR^Bglap^;R26^ZSG^ newborn mice (Fig. S3b–d). Collectively, the morphologic, immunostaining, strength, mineral apposition, and labeling data strongly suggest that SHP2 influences endochondral bone formation by regulating the differentiation, maturation, and function of osteoblastic cells, and SHP2 deficiency has a minimal effect on cell proliferation and viability but can induce adipogenic commitment of early osteoblastic cells.

### SHP2 deletion compromises osteoblastic-cell maturation and function

**Fig. 3:**
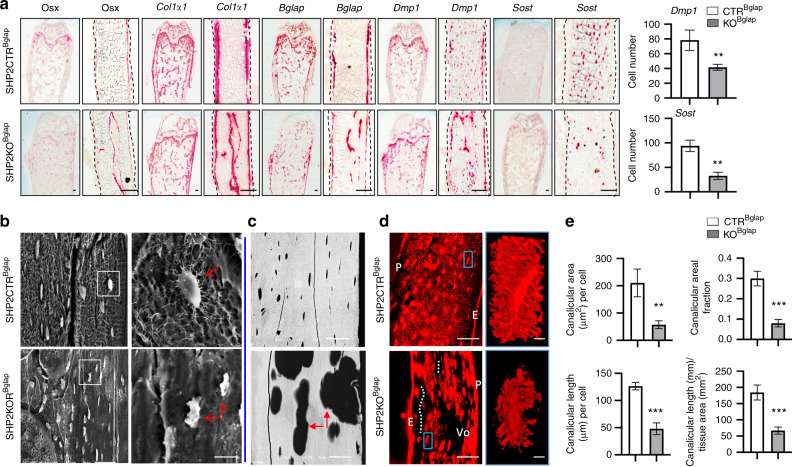
SHP2 regulates osteoblast maturation and function. **a** Images of femoral frozen sections show the transcript abundance of the indicated osteoblastic genes determined using RNAScope® assays (low-power images shown in Fig. S4a). *Dmp1*- and *Sost*-positive cells were quantified from the selected areas (500 × 200 μm) of the central diaphyseal cortical bone from the control and SHP2 mutants. Areas selected for enlarged view are shown in Fig. S4a. The quantified data are shown by bar graphs on the right (*n* = 3, **P < 0.01, Student’s *t*-test). Acid etching (**b**) and back-scattered SEM (**c**) images of the femoral cortical bone show the microarchitecture of osteocytes in 13-week-old mice with the indicated genotypes. Enlarged views of the boxed areas in **b** and **d** are shown in the right panel of each figure. Arrow, osteocytes, unmineralized matrix. **d** Confocal imaging showed a disorganized osteocyte lacunar–canalicular system (LCS, red) with disrupted areas (indicated by dotted lines and Vo) in the humeral cortex of 13-week-old mouse mutants. Enlarged views of the boxed areas are shown on the right. E endosteum, P periosteum, Vo void without canaliculi. **e** Impaired canalicular network (area or length per cell or per unit tissue) in mutants (*n* = 4, **P < 0.01, ***P < 0.001, Student’s *t*-test). Scale bar: **a** 100 μm; **b** 50 μm; **c** 50 μm; **d** 50 μm (left); 3 μm (right)

To understand how SHP2 modulates osteoblastic-cell differentiation, function and skeletal development, we first examined the effect of SHP2 deletion on the expression of marker genes from early- and late-stage developing OBs using RNAscope^®^-based in situ hybridization. Early osteoblastic cells express *Osx*, *Col1a1*, and *Bglap*, while terminally differentiated OBs and osteocytes express high levels of *Dmp1* and *Sost1*, which regulate matrix mineralization and the maintenance of mineral homeostasis.^48^ In SHP2CTR^Bglap^ mice, *Sost* and *Dmp1* were expressed at high levels in osteocytes of both cortical and trabecular bone. In contrast, significantly lower levels were expressed in the corresponding cells in SHP2KO^Bglap^ mutants (Figs 3a and S4, **P < 0.01, respectively). Consistent with these findings, *Sost* and *Dmp1* expression was significantly reduced in 13-week-old TM-treated SHP2KO^Bglap/ER^ mice compared to SHP2CTR^Bglap/ER^ mice (Fig. S5a). Importantly, high levels of the early osteoblastic-cell markers *Osx, Col1α1*, and *Bglap* were atypically expressed in the cortical bones of the SHP2KO^Bglap^ mutants (Figs 3a and S4). These data suggest that SHP2 is required for terminal OB maturation and that its deficiency is associated with persistent immaturity.

In compact bone, mature OBs lay down osteoids and transform into osteocytes embedded in the mineralized bone matrix. Mature osteocytes are morphologically and functionally networked together via their dendrites through the canaliculi to provide a coordinated response to matrix mineralization. To explore the role of SHP2 in osteocyte differentiation and maturation, we examined the morphology and function of osteocytes using acid-etched scanning electronic microscopy (SEM). Osteocytes from SHP2KO^Bglap^ mutants were markedly deformed with few, if any, dendrites compared to those of the SHP2CTR^Bglap^ controls (Fig. 3b). Back-scattered SEM (BSEM) revealed increased porosity in the cortical bone of SHP2KO^Bglap^ mutants, indicating that matrix production and mineralization were compromised as a consequence of osteocyte maturation and functional defects (Fig. 3c). The above observations were further supported by studies of the lacunar–canalicular system (LCS) in the cortex of the long bones. In the SHP2CTR^Bglap^ mice, osteocytes were uniformly distributed, and LCS interconnectivity was high, as revealed by 2D imaging of humerus sections (Figs 3d, top and S6c, d). In contrast, osteocytes were sparsely and irregularly distributed in SHP2KO^Bglap^ mutants, and the LCS was disrupted with shortened and disorganized dendrites (Figs 3d, bottom and S6c, d). Quantitative comparison of the density of the canalicular networks showed that they were significantly reduced in the bone cortex of SHP2 mutants (Fig. 3d, e). H&E and polyethylene glycol-associated solvent system (PEGASOS)-based imaging analysis^49^ revealed that the unmineralized areas were comprised of cells, matrix fibers, and holes (Fig. S6a, b). Collectively, these data suggest that SHP2 is essential for the terminal differentiation, maturation, and function of OBs and osteocytes and for bone matrix mineralization and that SHP2 depletion significantly decreases the number of dendrites and impairs canalicular network formation.

### SHP2 deletion in *Bglap*^+^ osteoblastic cells promotes RANKL expression and local osteoclastogenesis

**Fig. 4:**
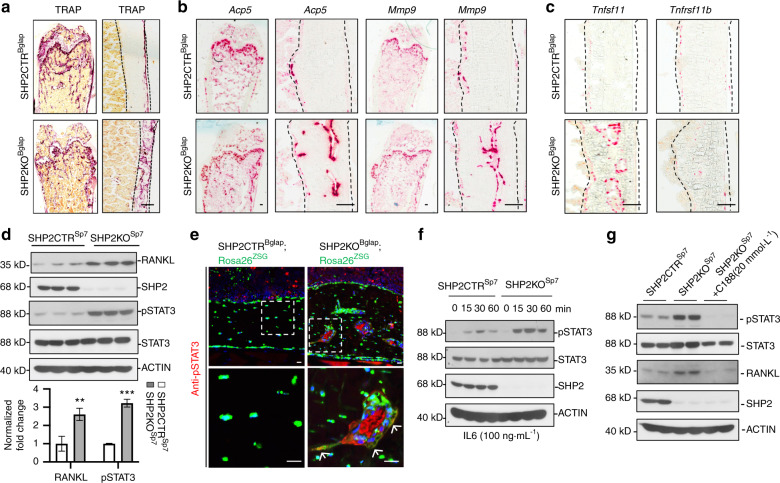
Mice lacking SHP2 in *Bglap* ^+^ osteoblastic cells display elevated local osteoclastogenesis and bone resorption. **a** Femoral frozen sections from the indicated 13-week-old mice show the activity of tartrate-resistant acid phosphatase (TRAP). Note the markedly increased TRAP activity in the cortical bone of SHP2 mutants compared to control mice. The quantitative data are provided in Fig. S2d. Scale bar: 100 μm. **b** Femoral frozen sections show the transcript abundance of the indicated osteoclastic genes in the metaphysis and diaphyseal cortical bone using RNAScope® technology (*n* = 3–4). Scale bar: 100 μm. **c** Femoral frozen sections show the transcript abundance of RANKL *(Tnfsf11)* and OPG *(Tnfrsf11b)* in the diaphyseal cortical bone using RNAScope® assays (*n* = 3). Scale bar: 100 μm. **d** Western blots show the expression of RANKL and SHP2 and the phosphorylation of STAT3 Y705 in osteoblast cell lines derived from the indicated mice. **e** Femoral frozen sections immunostained with antibodies against pSTAT3 Y705 demonstrate STAT3 activation in the OBs (arrows) (*n* = 4). Scale bar: 100 μm. **f** Western blots show the abundance and activation of STAT3 in SHP2-sufficient and SHP2-deficient OBs upon IL6 stimulation. **g** Western blots show the abundance of STAT3, SHP2, and RANKL in SHP2-sufficient and SHP2-deficient OBs in the presence or absence of the STAT3 inhibitor C188. In all western blotting studies, ACTIN served as an internal loading control. The low-power full images of Fig. 4a–c shown in Figs S2c and S4b

OBs and osteocytes are the most abundant cells in the adult skeleton. Beyond their primary role in bone formation, maintenance, and mineral homeostasis, they also influence osteoclastogenesis and osteoclast function via the synthesis and secretion of RANKL (*Tnfsf11*)^50–52^ and OPG (*Tnfrsf11b*).^27,53^ Importantly, osteocytes have been shown to be the predominant source of RANKL during bone remodeling, and osteocyte-derived RANKL is a critical mediator of bone resorption.^54,55^ Given the reduction in bone mass and appearance of porous cortical bone in SHP2KO^Bglap^ mice, we examined whether SHP2 deletion in *Bglap*^+^ cells affects local RANKL production and osteoclastogenesis. First, we screened for increases in the number of osteoclasts by staining for tartrate-resistant acid phosphatase (TRAP). As shown in Figs 4a and S2c, d, TRAP-stained frozen tibial sections revealed a significant increase in the number of osteoclasts in the metaphysis and cortical bone of SHP2KO^Bglap^ mice. The increase in TRAP staining was associated with elevated numbers of cells positive for *Acp5*, *Mmp9*, and, most importantly, *Tnfsf11* (RANKL) but not *Tnfrsf11b* (OPG) (Figs 4b, c and S4). These findings were corroborated by western blotting analysis, which demonstrated increased RANKL *(Tnfsf11)* in SHP2-deficient OBs (Fig. 4d). Collectively, these data indicate that under physiological conditions, SHP2 suppresses the production of RANKL by osteoblastic cells and that SHP2 deficiency can drive local osteoclastogenesis by promoting RANKL production, leading to mineral resorption and skeletal porosity.

### SHP2-modulated STAT3 activation is required for local RANKL production

Multiple signaling pathways regulate RANKL expression, particularly the activation of STAT3 (ref. ^50^). SHP2 has been reported to negatively regulate STAT3 activation.^56,57^ Our finding of increased *Tnfsf11* (RANKL) expression by SHP2 deletion in *Bglap*^+^ OBs (Fig. 4c) prompted us to investigate whether SHP2 modulates RANKL production by influencing STAT3 activation. To do so, we first generated OB cell lines from the calvaria of SHP2CTR^Sp7^ and SHP2KO^Sp7^ newborns using SV40 large T-cell-mediated immortalization (Fig. S7a, b). Western blotting analysis of these OB cell lines (Fig. 4d) and immunostaining of cortical bones (Fig. 4e) revealed that STAT3 Y705 phosphorylation was enhanced in SHP2-deficient OBs compared to that in controls. Moreover, in SHP2-deficient OBs, STAT3 Y705 phosphorylation was elevated in response to IL6, an inflammatory cytokine that is often upregulated and associated with osteolysis in rheumatoid arthritis patients (Fig. 4f). To provide additional confirmation, SHP2-deficient OBs were treated with the STAT3-specific inhibitor C188. Inhibition of STAT3 activation, as revealed by STAT3 Y705 phosphorylation, significantly decreased the level of RANKL (Fig. 4g). Collectively, these data indicate that STAT3 phosphorylation and activation are negatively regulated by SHP2 in OBs and osteocytes and that SHP2 modulates RANKL production in these cells by regulating STAT3 phosphorylation.

### OSTERIX is required for SHP2-mediated osteogenic gene expression

**Fig. 5:**
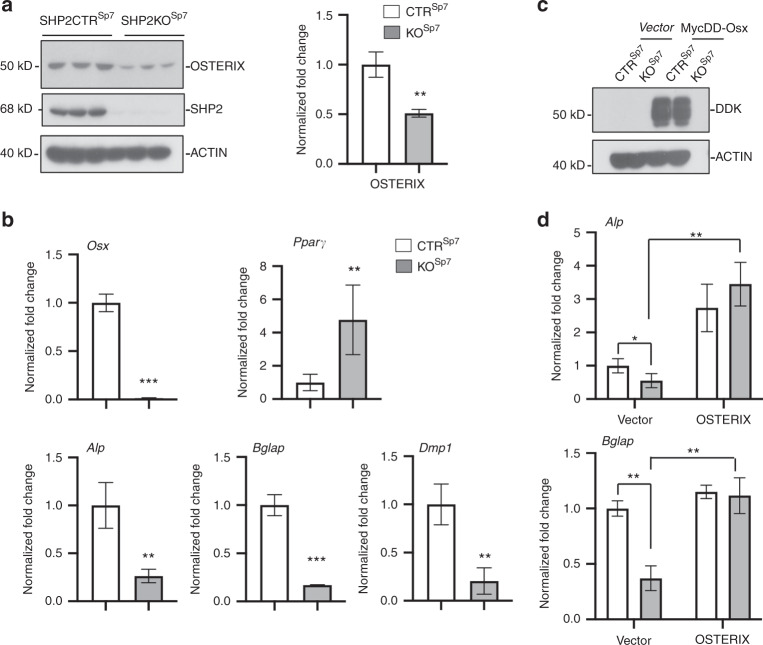
SHP2 regulates osteogenic differentiation by influencing OSTERIX expression. **a** Western blots show the expression level of OSTERIX in SHP2-sufficient and SHP2-deficient OBs, with quantitative data shown as bar graphs on the right. ACTIN served as an internal loading control. **b** Bar graphs show the transcript abundance of the indicated osteogenic and adipogenic genes in control and SHP2-deficient OBs determined by qRT-PCR (*n* = 3, **P < 0.01, ***P < 0.001, Student’s *t*-test). **c** Western blots show the expression of the vector and MycDD-OSTERIX in transiently transfected SHP2CTR^Sp7^ and SHP2KO^Sp7^ OBs. The transfection efficiency was evaluated by immunoblotting against the DDK tag. **d** Bar graphs show the transcript abundance of *Alp* and *Bglap* in transiently transfected SHP2CTR^Sp7^ and SHP2KO^Sp7^ OBs determined by qRT-PCR (*n* = 3, *P < 0.05, **P < 0.01, Student’s *t*-test)

To study the mechanism whereby SHP2 regulates OB maturation, we carried out co-immunoprecipitation, western blotting, and qRT-PCR analysis using SHP2-sufficient and SHP2-deficient OBs. Given the master regulatory role of RUNX2 and OSTERIX in OB differentiation and maturation and the suppression of the adipogenic commitment of mesenchymal progenitors,^58,59^ we first examined the effect of SHP2 deletion on the expression and abundance of other osteogenic and adipogenic genes. Although the RUNX2 level was comparable in SHP2-sufficient and SHP2-deficient OBs (data not shown), the abundance of OSTERIX, *Osx, Alp, Bglap*, and *Dmp1* was significantly reduced, but that of *Pparγ* was increased in SHP2-deficient OBs (Fig. 5a, b). Given the indispensable role of OSTERIX in OB differentiation and maturation and the marked reduction in OSTERIX in SHP2 mutant OBs, we reasoned that SHP2 might regulate OB and osteocyte maturation by influencing the expression of OSTERIX. To test this hypothesis, we transiently transfected SHP2-sufficient and SHP2-deficient OBs with MycDD-tagged OSTERIX (Fig. 5c) and then examined the expression of *Alp* and *Bglap* as an index of osteogenic differentiation. As shown in Fig. 5d, re-establishing the expression of OSTERIX restored the expression of both *Alp* and *Bglap* in SHP2-deficient OBs. These data strongly suggest that SHP2 modulates osteogenic and adipogenic differentiation, in part, by influencing the expression and activity of OSTERIX and *Pparγ*.

### SHP2 deletion in *Bglap*^+^ chondrocytes in mice leads to the growth of osteochondromas and multiple joint deformations

**Fig. 6:**
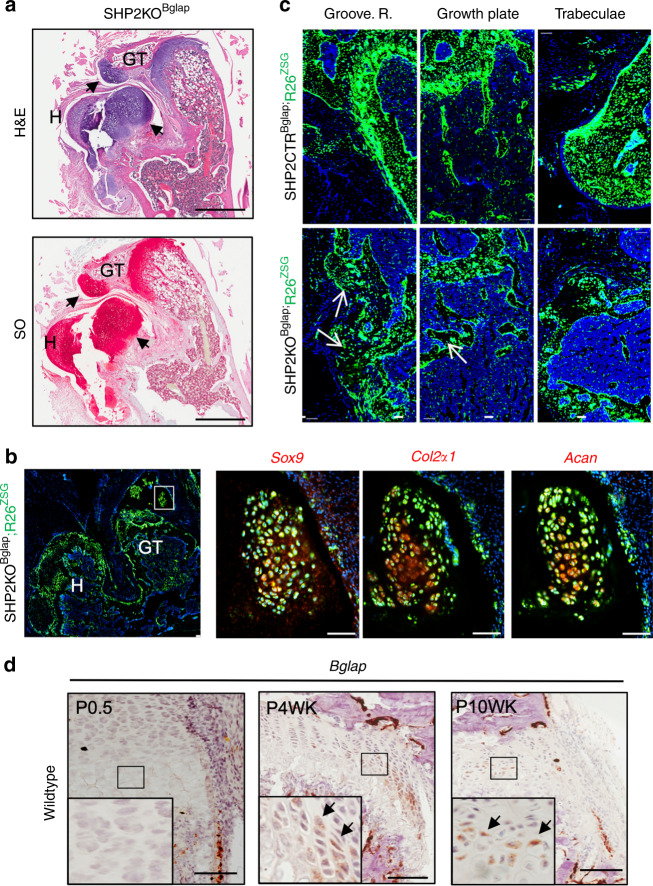
SHP2 deletion in *Bglap*^+^ chondroid cells leads to the growth of enchondromas and osteochondromas. **a** H&E- and SO-stained proximal femoral sections show the existence of cartilaginous enchondromas and osteochondromas in 13-week-old SHP2KO^Bglap^ mice. H femur head, GT great trochanter, Scale bar: 1 mm. **b** Proximal femoral frozen sections reveal enchondromas comprised of R26^ZSG+^ cells that also express *Sox9*, *Col2α1*, and *Acan*, as determined by RNAScope® assays. Scale bar: 100 μm. Green: R26^ZSG^ reporter; red: RNAScope pseudocolor for the indicated probes. **c** Frozen tibial sections exhibit R26^ZSG^-positive cells in the groove of Ranvier, growth plate, and trabeculae in 13-week-old controls and SHP2 mutants. Scale bar: 100 μm. **d** Frozen tibial sections show *Bglap* transcript abundance in the growth plate chondrocytes of postnatal 0.5-day-old, 4- and 10-week-old mice detected using RNAScope® assays. Enlarged view of the boxed areas shown at the bottom left. *Bglap*^+^ cells marked by arrows. Scale bar: 100 μm

Through 10 weeks of age, the SHP2CTR^Bglap^ and SHP2KO^Bglap^ mice were phenotypically indistinguishable. Shortly thereafter, the mutants progressively lost mobility and became incapacitated (please see the video clip in the SI). To understand the basis of the pathology, we performed histological analysis and in vivo live cell lineage tracing to determine the fate of the Bglap-expressing cells and their progeny. Interestingly, we found that the Bglap-driven R26^ZSG^ reporter lit up a large number of growth plate chondrocytes—in addition to OBs and osteocytes—in both the SHP2CTR^Bglap^;R26^ZSG^ and SHP2KO^Bglap^;R26^ZSG^ mice (Fig. S1f). Importantly, the number of R26^ZSG^ reporter-positive cells increased substantially in SHP2 mutants, forming osteochondromas and enchondromas (Fig. 6a–c) that express *Sox9*, *Col2a1*, and *Acan* (Figs 6b and S8), similar to those that develop in mice lacking SHP2 in *Col2a1*-expressing cells.^60,61^ To investigate the cellular origins of these tumors, we examined the temporal expression of *Bglap* in cartilage as a readout of *Bglap* promoter activity. As expected, *Bglap* mRNA was detected in growth plate chondrocytes in aged wild-type mice (Fig. 6d). Taken together, these data suggest that the growth of osteochondroma in SHP2KO^Bglap^;R26^ZSG^ mice results from the developmental activation of the *Bglap* promoter and the inactivation of SHP2 in chondroid but not osteoblastic cells and that this pathologic process is relatively slow, which is likely due to the low activity of *Bglap-Cre* in chondroid cells so that it cannot mediate efficient deletion of SHP2-floxed alleles.

**Fig. 7:**
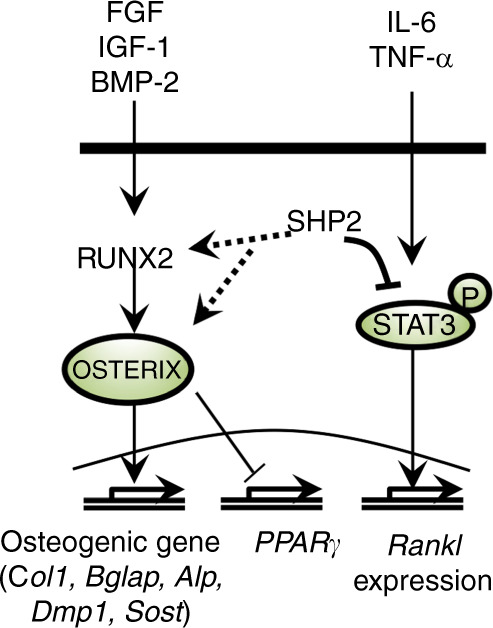
Diagrams demonstrate the molecular model by which SHP2 regulates osteoblast and osteocyte maturation and local osteoclastogenesis. SHP2 is physiologically required for osteoblast maturation by modulating the expression of OSTERIX and its responsive osteogenic genes and for local osteoclastogenesis regulation by repressing STAT3 phosphorylation and activation and RANKL expression, respectively

## Discussion

SHP2 is ubiquitously expressed and has been reported to have a cellular context-specific effect.^62^ SHP2 deletion in *Prrx1*^+^ osteochondroprogenitors favors chondrocytic differentiation,^44^ and SHP2 deletion in *Col2α1* chondrocytes promotes cell proliferation and cartilage tumor formation that mimics human MC.^60,63^ While few studies regarding the role of SHP2 in bone cells have been carried out, we found that mice with SHP2 deletion in *Bglap*-expressing cells displayed a spectrum of skeletal abnormalities, including defective ossification, the appearance of a porous skeleton and excessive adipocytes in the bone marrow cavity, decreased BV, age-related growth plate cartilage expansion, and the growth of cartilaginous lesions that cause joint deformations (Figs 1–3). Although body lengths were comparable between the control and SHP2KO^Bglap^ mutants, tubular bone length increased significantly as a consequence of altered growth plate cartilage and the discrete organization and growth control of the axial and appendicular skeletons. These findings strongly suggest that SHP2 is required for normal differentiation and function of OBs and osteocytes and for cartilage and joint homeostasis. Indeed, our mechanistic studies revealed that the number of mature OBs and osteocytes was significantly reduced in mice in which SHP2 was deleted in Bglap-expressing cells, as evidenced by the decrease in the number of *Dmp1-* and *Sost*-positive cells, decreases in both the number and length of osteocyte dendrites in developing and adult SHP2KO^Bglap^ mice (Figs 3 and S5), and compromised osteogenic gene expression (Fig. 5). Importantly, the above changes were also associated with a significant increase in tubular bone length (Fig. S2a) and local osteoclastogenesis (Fig. 4), and the restoration of OSTERIX expression in SHP2-deficient OBs and pharmacological inhibition of STAT3 activation restored osteogenic gene expression and normal RANKL production, respectively (Figs 4 and 5). Together, these data indicate that SHP2 is essential to support osteogenesis and bone mineral homeostasis by regulating the differentiation, maturation, and function of OBs and osteocytes and by repressing RANKL production and local osteoclastogenesis.

The resemblance of the skeletal phenotypes in SHP2KO^Bglap^, RUNX2, and OSTERIX knockout mice suggests that SHP2 may signal through these molecules and their modulated signaling pathways.^2,3,64^ RUNX2 and OSTERIX are essential for osteoblastogenesis and skeletal development. Although global RUNX2 deletion in mice causes perinatal lethality,^65,66^ inducible RUNX2 deletion in adult mice leads to decreased *Osx* expression, reduced BV, and excessive BM adipocyte accumulation.^67^ These features are recapitulated in SHP2KO^Bglap^ mice. Therefore, we investigated the interaction of SHP2 with RUNX2 and its effect on RUNX2 tyrosyl phosphorylation. Indeed, SHP2 was found to physically interact with RUNX2 in OBs, but its deficiency had no apparent influence on RUNX2 global tyrosyl phosphorylation (data not shown). Our study, however, did not definitively exclude regulation of RUNX2 phosphorylation by SHP2, since 15 putative tyrosyl phosphorylation sites and multiple serine phosphorylation sites have been described in the literature. The lack of site-specific antibodies currently hinders this investigation. OSTERIX, which acts downstream of RUNX2, is a zinc finger-containing transcription factor predominantly expressed in OB lineage cells.^2^ OSTERIX is essential for the activation of a host of genes involved in OB differentiation; its deficiency arrests OB and osteocyte maturation and new bone formation.^64^ Indeed, SHP2 deletion significantly downregulated the level of OSTERIX and its responsive genes *Alp* and *Bglap* (Figs 3 and 5). Overexpression of OSTERIX in SHP2 mutant OBs restores osteoblastic gene (e.g., *Alp* and *Bglap*) expression (Fig. 5), suggesting that SHP2 regulates osteoblastogenesis and osteocyte maturation likely by influencing the RUNX2-OSTERIX signaling axis. Although the exact molecular mechanism by which SHP2 modulates OSTERIX and *Osx* expression remains unclear, SHP2 has been reported to modulate the activation of RUNX family transcription factors^68^ and SRC family kinases,^69^ both of which can influence OSTERIX expression and activity.^9,70^ Studies are ongoing to delineate how SHP2 modifies the expression and activity of OSTERIX.

Studying the skeletal phenotype of SHP2KO^Bglap^ mice has led to the discovery of SHP2-mediated OB regulation of local osteoclastogenesis. While reciprocal communication between osteoclasts and OBs via the RANKL/RANK pathway has been previously described,^54,71^ the underlying molecular mechanism has yet to be described. Our findings that the abundance of RANKL and osteoclastogenic genes and the activity of TRAP were significantly increased in SHP2KO^Bglap^ mice and SHP2-deficient OBs (Fig. 4) indicate that SHP2 in osteoblastic cells regulates local osteoclastogenesis and that this regulation is mediated by elevated STAT3 activation (Fig. 4). Our findings are consistent with published work showing that SHP2 downregulates STAT3 phosphorylation^72,73^ and that mice bearing *gp130F759/F759* mutations that no longer bind SHP2 have enhanced STAT3 activation and osteoclast formation and consequently BMD reduction.^74,75^ STAT3 is essential for gp130-mediated osteoclast formation by inducing the production of RANKL.^50^ Indeed, IL6-evoked STAT3 activation was enhanced in OBs lacking SHP2 (Fig. 4). Moreover, STAT3 mutations cause a rare human immunodeficiency disease, Job’s syndrome, featuring reduced BMD, recurrent fractures, and scoliosis.^76^ These published data are consistent with the phenotypes observed in SHP2KO^Bglap^ mice, such as a porous skeleton and reduced BMD, and indicate that signaling along the SHP2/STAT3 axis in OBs is critical for osteoclastogenesis and the maintenance of bone mineral homeostasis.

The finding of osteochondroma and enchondroma growth in SHP2KO^Bglap^ mice extends our understanding of the dynamics of *Bglap* promoter activity in vivo and of the regulatory role of SHP2 in cartilage. It has been reported that the *Bglap* promoter is active in osteogenic cells and that its activity in these cells declines with aging.^77^ However, the dynamics of *Bglap* promoter activity in cartilage are less well-studied and remain unclear. Our lineage tracing and in situ hybridization studies demonstrate that the *Bglap* transcript was undetectable in perinatal growth plate chondrocytes, but its abundance progressively increased with age. The delayed cartilage tumor onset and growth and the expanded growth plate cartilage in old SHP2KO^Bglap^ mice could be explained by the relatively weak activity *of Bglap*-Cre, which would prolong the time needed to excise both SHP2-floxed alleles, in contrast to the robust activity of *Col2α1-Cre* and *Ctsk-Cre* in cartilage.^60,63^ The time lag for osteochondroma development could also reflect the time required for mutations in other genes to occur and manifest themselves in benign tumor formation. The enchondromas and osteochondromas found in old SHP2KO^Bglap^ mice were primarily comprised of R26^ZSG+^ (*Bglap*^*+*^) cells that also expressed *Sox9*, *Col2α1*, and *Acan* (Figs 6b, c and S8), mimicking the features of human MC in which somatic *PTPN11* LOF mutations and enhanced chondrocytic gene expression have been identified.^39,40^ This notion supports the tumor suppressor role of SHP2 in cartilage^39,40^ and suggests that BGLAP may play a role in growth plate cartilage during aging.

In summary, our study expands on the crucial role of SHP2 in regulating the differentiation, maturation, and function of cells in the OB lineage, likely by modifying the RUNX2/OSTERIX signaling axis in mature OBs and maintaining local osteoclastogenesis and bone mineral homeostasis by modifying STAT3 activation (Fig. 7). In contrast to the role of SHP2 in repressing SOX9 expression and the chondrogenic commitment of *Prrx1*^+^ osteochondroprogenitors, SHP2 is required for the expression of the master osteogenic transcription factor OSTERIX to ensure cellular differentiation along the osteogenic lineage. SHP2 deletion in *Bglap*^+^ osteoblastic cells diverts their differentiation toward the adipogenic but not the chondrogenic lineage, reinforcing the idea that SHP2 has a developmental stage-specific effect in bone cells and that early *Bglap*^+^ osteoblastic cells remain bipotent. The ability to manipulate the development and function of *Bglap*^+^ osteogenic cells and local osteoclastogenesis by modulating SHP2 activity suggests a new strategy to combat OB-related disorders, such as osteoporosis.

## Materials and methods

### Transgenic mice

*Ptpn11*-floxed (*Ptpn11*^*fl*^) mice,^39^ *Tg(Bglap-Cre)*,^45^ *Tg(Bglap-CreER)*,^46^ *Tg(Sp7-Cre), and Tg(Rosa26*^*ZsG*^*)*^47^ mice were reported previously. PCR genotyping conditions for the *Ptpn11*-floxed and *Rosa26*^*ZsG*^ alleles and the Cre transgenes are described in the original publications and are available upon request. To induce *Tg(Bglap-CreER)* activity in vivo and in vitro, tamoxifen was administered to mice or added to the culture medium as described previously.^44,60^ Control and SHP2 mutants were sacrificed at the indicated time points and underwent X-ray, µ-CT, histological, biochemical, and biological analyses. All transgenic mice were maintained on the C57BL/6 background and studied in accordance with the Institutional Animal Care and Use Committee approved protocols.

### OB culture and exogenous gene expression

Primary OBs were isolated from 0.5-day-old pups as described.^78^ Briefly, calvarial bones were collected under sterile conditions and incubated in digestion solution (0.06% trypsin-EDTA and 285 U·mL^−1^ collagenase II) for 2 h at 37 °C. The digestion was neutralized by DMEM with 10% FBS and passed through a 40-µm cell strainer to obtain single cells. After washing with PBS twice, OBs were collected by centrifugation and cultured in DMEM (Invitrogen) supplemented with 10% FBS and 1% ampicillin and streptomycin (Invitrogen). After 2–3 passages, GFP^+^ OBs were enriched by FACS for biological and biochemical studies or immortalized with SV40 large T antigen to establish cell lines as previously described.^44^

### DNA constructs, transfection, and lentiviral infection

pBABE(Neo)SV40LT and pCMV6-MycDD-OSTERIX plasmids were obtained from Addgene and Origene, respectively. Packaging of retrovirus and infection of osteoblastic cells followed the manufacturer’s instructions (Clontech). Fugene (Promega) was used for transient transfection of 293T cells and OBs.

### Antibodies and reagents

Polyclonal and monoclonal antibodies (PcAb and McAb) were purchased from commercial sources: McAb against phospho(p)-STAT3 (Y705), STAT3 was purchased from Cell Signaling Inc., and McAb against DDK was purchased from OriGene. PcAbs against SHP2 were purchased from Santa Cruz Inc., and PcAbs against OSTERIX, RANKL, and ACTIN were purchased from Abcam Inc. EdU labeling and detection kits were obtained from Life Technologies (NJ). Inhibitors of STAT3 (c188) were purchased from Selleckchem (TX). The goat anti-rabbit IgG secondary antibody Alexa Fluor 594 was purchased from Invitrogen Inc. ALP, von Kossa, safranin O, H&E, xylene orange, and TRAP kits were from Aldrich-Sigma (MO). IL6 cytokine was purchased from PeproTech Inc.

### Histological analysis

To examine the effect of SHP2 deletion in *Bglap*^+^ cells on gross skeletal development, mice were eviscerated after euthanasia and fixed in 4% paraformaldehyde (PFA) overnight or for 2–3 days depending on the body size at 4 °C. For H&E staining to examine the gross histology, tibiae or femurs were fixed and decalcified before sectioning. For von Kossa, Safranin O, ALP, TRAP, and antibody staining, 7-µm-thick frozen sections of femurs and tibiae without decalcification were used. To analyze the calcified bone tissue per von Kossa staining, a 1 mm by 1.2-mm region beneath the proximal tibia growth plate cartilage was selected and quantified using NIH ImageJ software. To trace the fate of *Bglap*^+^ OBs in vivo, frozen femur and tibia sections of postnatal (P) 0.5-day-old and 4 to 10-week-old mice were examined microscopically. DAPI was used to counterstain the nucleus. All fluorescent and phase-contrast images were obtained using a Nikon digital fluorescence microscope and an Aperio slide scanner (Vista, CA). Immunostaining of tissue sections was carried out using a goat anti-rabbit secondary antibody, Alexa Fluor 594, following the manufacturer’s instructions.

### µ-CT and X-ray radiograph analysis

Plain radiography of mouse skeletons was carried out immediately after euthanasia using a high-resolution digital cabinet X-ray system (MX-20, Faxitron Bioptics, LLC, Tucson, USA). High-resolution 3-D volume images of fixed skeletal elements were generated using a desktop µ-CT40 system (Scanco Medical AG, CH).

### EM analysis of osteocytes

For resin-casted SEM, dissected long bones were fixed in 4% PFA for 24 h. The specimens were dehydrated, embedded in methyl methacrylate, and then surface polished as described.^64^ The bone surface was acid-etched with 37% phosphoric acid for 2–10 s, followed by treatment with 5% sodium hypochlorite for 5 min. The samples were then coated with gold and palladium and examined using an FEI/Philips XL30 Field-Emission Environmental SEM. For BSEM, the long bones were fixed overnight in 2% PFA and 2% glutaraldehyde buffered at pH 7.4 with 0.1 mol·L^−1^ sodium cacodylate. Samples were then rinsed three times (20 min each time) in 0.1 mol·L^−1^ cacodylate buffer solution followed by secondary fixation (1 h) in a solution of 1% osmium tetroxide in 0.1 mol·L^−1^ cacodylate buffer. The BSEM samples were then coated with carbon and examined using an FEI/Philips XL30 Field-Emission Environmental SEM.

### In situ hybridization and quantitative RT-PCR analyses

Femoral and tibial frozen sections (7 µm) were collected from mice sacrificed at the indicated time points and used for in situ hybridization with probes against murine *Acp5, Sp7, Col1α1, Bglap, Dmp1, Sost1, Runx2, Mmp9, Tnfsf11, Tnfrsf11b, Ibsp, Col2α1, Col10α1, Pthr1*, and *Mmp13*. Hybridization and detection of hybridization signals were achieved using the RNAScope® HD-Red and Brown kits per the manufacturer’s instructions (Advanced Cell Diagnostics) and evaluated using NIH ImageJ software.

To measure the abundance of osteogenic stage-specific marker gene expression, total RNA was extracted from FACS-enriched GFP^+^ OBs using an RNeasy kit (Qiagen). qRT-PCR was performed with an RT^2^SYBR®Green qRT-PCR kit on a Bio-Rad CFX machine using cDNA that was synthesized using 1 µg total RNA with an iScript™cDNA Synthesis Kit (Bio-Rad). All samples were normalized to *Actin*, and gene expression data are presented as fold increases or fold decreases compared to controls. The primer sequences used for this study are listed in Fig. S9.

### Immunoprecipitation and western blot analysis

Cells were lysed in modified NP40 lysis buffer [0.5% NP40, 150 mmol·L^−1^ NaCl, 1 mmol·L^−1^ EDTA, 50 mmol·L^−1^ Tris (pH 7.4)] supplemented with a protease inhibitor cocktail (1 mmol·L^−1^ PMSF, 10 mg·mL^−1^ aprotinin, 0.5 mg·mL^−1^ antipain, and 0.5 mg·mL^−1^ pepstatin). For immunoblotting, cell lysates (30–50 µg) were resolved by SDS-PAGE, transferred to PVDF membranes, and incubated with primary antibodies for 2 h or overnight at 4 °C (according to the manufacturer’s instructions), followed by incubation with HRP-conjugated secondary antibodies (Bio-Rad).

### PEGASOS tissue clearing and 3-D image acquisition

PEGASOS tissue clearing treatment was performed as previously described.^49^ In brief, after perfusion, long bones were fixed in 4% PFA. Decalcification was carried out in 10% EDTA. Decolorization treatment was performed by shaking samples in 25% Quadrol (Sigma-Aldrich 122262). Delipidation treatment was performed in gradient, 30%, 50%, and 70% tert-butanol aqueous solutions (Sigma-Aldrich 360538). Dehydration treatment was performed with tB-PEG solution. For clearing, samples were immersed in the BB-PEG clearing solution until complete transparency was achieved in ~24 h. 3-D imaging was performed with a 20 × 0.85 NA objective on a Zeiss LSM780 2-photon microscope. 3-D image rendering was performed with Imaris 9.0.

### Statistical analysis

Statistical differences between groups were evaluated by Student’s *t*-test analysis. *P* values of <0.05 were considered significant. Analyses were performed using Prism 6.0 (GraphPad, San Diego, CA) and Excel.

## Supplementary information

Supplementary Information

Video Clip

## References

[1] Nishimura R (2012). Osterix regulates calcification and degradation of chondrogenic matrices through matrix metalloproteinase 13 (MMP13) expression in association with transcription factor Runx2 during endochondral ossification. J. Biol. Chem..

[2] Nakashima K (2002). The novel zinc finger-containing transcription factor osterix is required for osteoblast differentiation and bone formation. Cell.

[3] Ducy P, Zhang R, Geoffroy V, Ridall AL, Karsenty G (1997). Osf2/Cbfa1: a transcriptional activator of osteoblast differentiation. Cell.

[4] Huang W, Zhou X, Lefebvre V, de Crombrugghe B (2000). Phosphorylation of SOX9 by cyclic AMP-dependent protein kinase A enhances SOX9’s ability to transactivate a Col2a1 chondrocyte-specific enhancer. Mol. Cell Biol..

[5] Liu JA (2013). Phosphorylation of Sox9 is required for neural crest delamination and is regulated downstream of BMP and canonical Wnt signaling. Proc. Natl Acad. Sci. USA..

[6] Vimalraj S, Arumugam B, Miranda PJ, Selvamurugan N (2015). Runx2: structure, function, and phosphorylation in osteoblast differentiation. Int. J. Biol. Macromol..

[7] Wee HJ, Huang G, Shigesada K, Ito Y (2002). Serine phosphorylation of RUNX2 with novel potential functions as negative regulatory mechanisms. EMBO Rep..

[8] Ortuno MJ (2010). p38 regulates expression of osteoblast-specific genes by phosphorylation of osterix. J. Biol. Chem..

[9] Choi YH (2015). Src enhances osteogenic differentiation through phosphorylation of Osterix. Mol. Cell Endocrinol..

[10] Maupin KA, Droscha CJ, Williams BO (2013). A comprehensive overview of skeletal phenotypes associated with alterations in Wnt/beta-catenin signaling in humans and mice. Bone Res..

[11] Day TF, Guo X, Garrett-Beal L, Yang Y (2005). Wnt/beta-catenin signaling in mesenchymal progenitors controls osteoblast and chondrocyte differentiation during vertebrate skeletogenesis. Dev. Cell.

[12] Burns, K. A. & Vanden Heuvel, J. P. Modulation of PPAR activity via phosphorylation. *Biochim. Biophys. Acta* **1771**, 952–960 (2007). 10.1016/j.bbalip.2007.04.018 PMC2712836 17560826

[13] Brunmeir, R. & Xu, F. Functional regulation of PPARs through post-translational modifications. *Int. J. Mol. Sci.* **19**, 1738 (2018). 10.3390/ijms19061738 PMC6032173 29895749

[14] Kawane T (2018). Runx2 is required for the proliferation of osteoblast progenitors and induces proliferation by regulating Fgfr2 and Fgfr3. Sci. Rep..

[15] Komori T (2010). Regulation of osteoblast differentiation by Runx2. Adv. Exp. Med Biol..

[16] Narayanan K (2003). Dual functional roles of dentin matrix protein 1. Implications in biomineralization and gene transcription by activation of intracellular Ca^2+^ store. J. Biol. Chem..

[17] Poole KE (2005). Sclerostin is a delayed secreted product of osteocytes that inhibits bone formation. FASEB J..

[18] Feng JQ (2006). Loss of DMP1 causes rickets and osteomalacia and identifies a role for osteocytes in mineral metabolism. Nat. Genet..

[19] Winkler DG (2003). Osteocyte control of bone formation via sclerostin, a novel BMP antagonist. EMBO J..

[20] Balemans W (2001). Increased bone density in sclerosteosis is due to the deficiency of a novel secreted protein (SOST). Hum. Mol. Genet..

[21] Mundlos S (1997). Mutations involving the transcription factor CBFA1 cause cleidocranial dysplasia. Cell.

[22] Lapunzina P (2010). Identification of a frameshift mutation in Osterix in a patient with recessive osteogenesis imperfecta. Am. J. Hum. Genet..

[23] Long F, Ornitz DM (2013). Development of the endochondral skeleton. Cold Spring Harb. Perspect. Biol..

[24] Bonewald LF (2011). The amazing osteocyte. J. Bone Min. Res..

[25] Graves DT, Jiang Y, Valente AJ (1999). Regulated expression of MCP-1 by osteoblastic cells in vitro and in vivo. Histol. Histopathol..

[26] Lacey DL (2012). Bench to bedside: elucidation of the OPG-RANK-RANKL pathway and the development of denosumab. Nat. Rev. Drug Discov..

[27] Boyce BF, Xing L (2008). Functions of RANKL/RANK/OPG in bone modeling and remodeling. Arch. Biochem. Biophys..

[28] Hayashi M (2012). Osteoprotection by semaphorin 3A. Nature.

[29] Franzoso G (1997). Requirement for NF-kappaB in osteoclast and B-cell development. Genes Dev..

[30] Takayanagi H (2002). RANKL maintains bone homeostasis through c-Fos-dependent induction of interferon-beta. Nature.

[31] Takayanagi H (2002). Induction and activation of the transcription factor NFATc1 (NFAT2) integrate RANKL signaling in terminal differentiation of osteoclasts. Dev. Cell.

[32] Aoki S (2010). Function of OPG as a traffic regulator for RANKL is crucial for controlled osteoclastogenesis. J. Bone Min. Res..

[33] Kobayashi Y, Udagawa N, Takahashi N (2009). Action of RANKL and OPG for osteoclastogenesis. Crit. Rev. Eukaryot. Gene Expr..

[34] Neel, B. G., Chan, G. & Dhanji, S. SH2 domain-containing protein-tyrosine phosphatases. *Handbook of Cell Signaling*, 771–809 (2009).

[35] Tartaglia M (2001). Mutations in PTPN11, encoding the protein tyrosine phosphatase SHP-2, cause Noonan syndrome. Nat. Genet..

[36] Araki T (2004). Mouse model of Noonan syndrome reveals cell type- and gene dosage-dependent effects of Ptpn11 mutation. Nat. Med..

[37] Legius E (2002). PTPN11 mutations in LEOPARD syndrome. J. Med. Genet..

[38] Yang W, Neel BG (2013). From an orphan disease to a generalized molecular mechanism: PTPN11 loss-of-function mutations in the pathogenesis of metachondromatosis. Rare Dis..

[39] Yang W (2013). Ptpn11 deletion in a novel progenitor causes metachondromatosis by inducing hedgehog signalling. Nature.

[40] Bowen ME (2011). Loss-of-function mutations in PTPN11 cause metachondromatosis, but not Ollier disease or Maffucci syndrome. PLoS Genet..

[41] Sobreira NL (2010). Whole-genome sequencing of a single proband together with linkage analysis identifies a Mendelian disease gene. PLoS Genet..

[42] Mansukhani A, Bellosta P, Sahni M, Basilico C (2000). Signaling by fibroblast growth factors (FGF) and fibroblast growth factor receptor 2 (FGFR2)-activating mutations blocks mineralization and induces apoptosis in osteoblasts. J. Cell Biol..

[43] Wang L (2019). SHP2 regulates intramembranous ossification by modifying the TGFbeta and BMP2 signaling pathway. Bone.

[44] Zuo C (2018). SHP2 regulates skeletal cell fate by modifying SOX9 expression and 1 transcriptional activity. Bone Res..

[45] Zhang M (2002). Osteoblast-specific knockout of the insulin-like growth factor (IGF) receptor gene reveals an essential role of IGF signaling in bone matrix mineralization. J Biol. Chem..

[46] Park D (2012). Endogenous bone marrow MSCs are dynamic, fate-restricted participants in bone maintenance and regeneration. Cell Stem Cell.

[47] Madisen L (2010). A robust and high-throughput Cre reporting and characterization system for the whole mouse brain. Nat. Neurosci..

[48] Nakashima K, de Crombrugghe B (2003). Transcriptional mechanisms in osteoblast differentiation and bone formation. Trends Genet..

[49] Jing D (2018). Tissue clearing of both hard and soft tissue organs with the PEGASOS method. Cell Res..

[50] O’Brien CA, Gubrij I, Lin SC, Saylors RL, Manolagas SC (1999). STAT3 activation in stromal/osteoblastic cells is required for induction of the receptor activator of NF-kappaB ligand and stimulation of osteoclastogenesis by gp130-utilizing cytokines or interleukin-1 but not 1,25-dihydroxyvitamin D3 or parathyroid hormone. J. Biol. Chem..

[51] Prideaux M, Findlay DM, Atkins GJ (2016). Osteocytes: the master cells in bone remodelling. Curr. Opin. Pharm..

[52] Atkins GJ (2003). RANKL expression is related to the differentiation state of human osteoblasts. J. Bone Min. Res..

[53] Udagawa N (2000). Osteoprotegerin produced by osteoblasts is an important regulator in osteoclast development and function. Endocrinology.

[54] Nakashima T (2011). Evidence for osteocyte regulation of bone homeostasis through RANKL expression. Nat. Med.

[55] Xiong J (2011). Matrix-embedded cells control osteoclast formation. Nat. Med..

[56] Zehender A (2018). The tyrosine phosphatase SHP2 controls TGFbeta-induced STAT3 signaling to regulate fibroblast activation and fibrosis. Nat. Commun..

[57] Cha Y, Park KS (2010). SHP2 is a downstream target of ZAP70 to regulate JAK1/STAT3 and ERK signaling pathways in mouse embryonic stem cells. FEBS Lett..

[58] Han Y, Kim CY, Cheong H, Lee KY (2016). Osterix represses adipogenesis by negatively regulating PPARgamma transcriptional activity. Sci. Rep..

[59] Enomoto H (2004). Runx2 deficiency in chondrocytes causes adipogenic changes in vitro. J. Cell Sci..

[60] Wang L (2017). SHP2 regulates the osteogenic fate of growth plate hypertrophic chondrocytes. Sci. Rep..

[61] Bowen ME, Ayturk UM, Kurek KC, Yang W, Warman ML (2014). SHP2 regulates chondrocyte terminal differentiation, growth plate architecture and skeletal cell fates. PLoS Genet..

[62] Chan, G. & Neel, B. G. Role of PTPN11 (SHP2) in cancer. in *Protein Tyrosine Phosphatases in Cancer In Protein Tyrosine Phosphatases in Cancer* (eds Neel, B. G. & Tonks, N. K.) 115–143 (Springer, New York, 2016).

[63] Kim, H. K., Feng, G. S., Chen, D., King, P. D. & Kamiya, N. Targeted disruption of Shp2 in chondrocytes leads to metachondromatosis with multiple cartilaginous protrusions. *J. Bone Miner. Res*. **29**, 761–769 (2014). 10.1002/jbmr.2062 PMC4081537 23929766

[64] Zhou X (2010). Multiple functions of Osterix are required for bone growth and homeostasis in postnatal mice. Proc. Natl Acad. Sci. USA..

[65] Komori T (1997). Targeted disruption of Cbfa1 results in a complete lack of bone formation owing to maturational arrest of osteoblasts. Cell.

[66] Otto F (1997). Cbfa1, a candidate gene for cleidocranial dysplasia syndrome, is essential for osteoblast differentiation and bone development. Cell.

[67] Tosa I (2019). Postnatal Runx2 deletion leads to low bone mass and adipocyte accumulation in mice bone tissues. Biochem. Biophys. Res. Commun..

[68] Huang H (2012). A Src family kinase-Shp2 axis controls RUNX1 activity in megakaryocyte and T-lymphocyte differentiation. Genes Dev..

[69] Zhang SQ (2004). Shp2 regulates SRC family kinase activity and Ras/Erk activation by controlling Csk recruitment. Mol. Cell.

[70] Adhami MD (2015). Loss of Runx2 in committed osteoblasts impairs postnatal skeletogenesis. J. Bone Min. Res..

[71] Ikebuchi Y (2018). Coupling of bone resorption and formation by RANKL reverse signalling. Nature.

[72] Zhang W (2009). Negative regulation of Stat3 by activating PTPN11 mutants contributes to the pathogenesis of Noonan syndrome and juvenile myelomonocytic leukemia. J. Biol. Chem..

[73] Ohtani T (2000). Dissection of signaling cascades through gp130 in vivo: reciprocal roles for STAT3- and SHP2-mediated signals in immune responses. Immunity.

[74] Naka T, Kishimoto T (2002). Joint disease caused by defective gp130-mediated STAT signaling. Arthritis Res..

[75] Atsumi T (2002). A point mutation of Tyr-759 in interleukin 6 family cytokine receptor subunit gp130 causes autoimmune arthritis. J. Exp. Med..

[76] Bergerson JRE, Freeman AF (2019). An update on syndromes with a hyper-IgE phenotype. Immunol. Allergy Clin. North Am..

[77] Frenkel B (1997). Activity of the osteocalcin promoter in skeletal sites of transgenic mice and during osteoblast differentiation in bone marrow-derived stromal cell cultures: effects of age and sex. Endocrinology.

[78] Bakker AD, Klein-Nulend J (2012). Osteoblast isolation from murine calvaria and long bones. Methods Mol. Biol..

