# Supplementary material for: Targeted Ptpn11 deletion in mice reveals the essential role of SHP2 in osteoblast differentiation and skeletal homeostasis
Source: Bone Res. 2021 Jan 27;9:6. doi: 10.1038/s41413-020-00129-7 (PMC7838289; doi:10.1038/s41413-020-00129-7)
Supplement: Supplementary file 1 — Supplementary Information [file 41413_2020_129_MOESM1_ESM.pdf]

Supplementary Information:

Targeted *Ptpn11* Deletion in Mice Reveals SHP2's Essential Role in Osteoblast Differentiation and Skeletal Homeostasis

Lijun Wang[1,6], Huiliang Yang[1,6], Jiahui Huang[1], Shaopeng Pei[2], Liyun Wang[2], Jian Q Feng[3], Dian Jing[4], Hu Zhao[4], Henry M. Kronenberg[5], Douglas C. Moore[1], and Wentian Yang[1]✉

[1]Department of Orthopaedic Surgery, Brown University Alpert Medical School and Rhode Island Hospital, Providence, RI 02903, USA. [2]Department of Mechanical Engineering, University of Delaware, Newark, DE19716. [3]Department of Biomedical Sciences and [4]Department of Comprehensive Dentistry, Texas A&M College of Dentistry, 3302 Gaston Ave, Dallas, TX 75246, USA. [5]Endocrine Unit, Massachusetts General Hospital and Harvard Medical School, Boston, MA 02114, USA. [6]These authors contributed equally: Lijun Wang and Huiliang Yang.

✉Corresponding Author:
Wentian Yang, M.D., Ph.D.
1 Hoppin Street, Coro 402E
Providence, RI 02903
401-4445956 (Phone)
wentian_yang@brown.edu

Running Title: SHP2 regulates osteoblast maturation and RANKL production

a

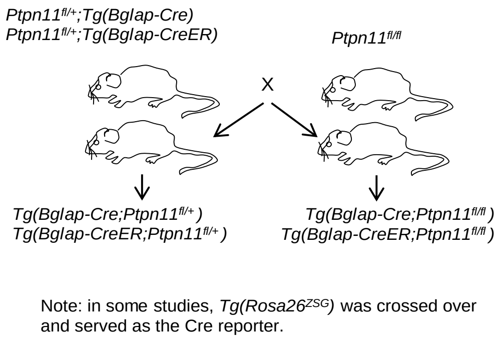

b

Tg(Bglap-Cre;Ptpn11$^{fl/+}$)	SHP2CTR^{Bglap}
Tg(Bglap-Cre;Ptpn11$^{fl/fl}$)	SHP2KO^{Bglap}
Tg(Bglap-CreER;Ptpn11$^{fl/+}$)	SHP2CTR$^{Bglap/ER}$
Tg(Bglap-CreER;Ptpn11$^{fl/fl}$)	SHP2KO$^{Bglap/ER}$

c
d

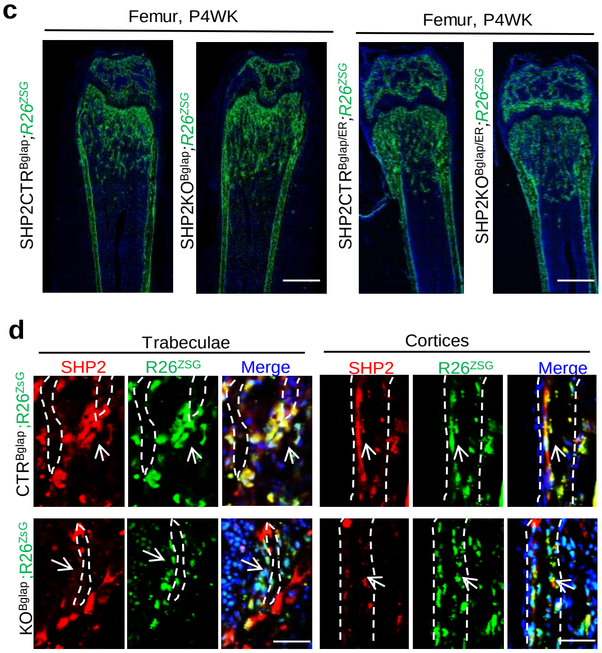

e
f

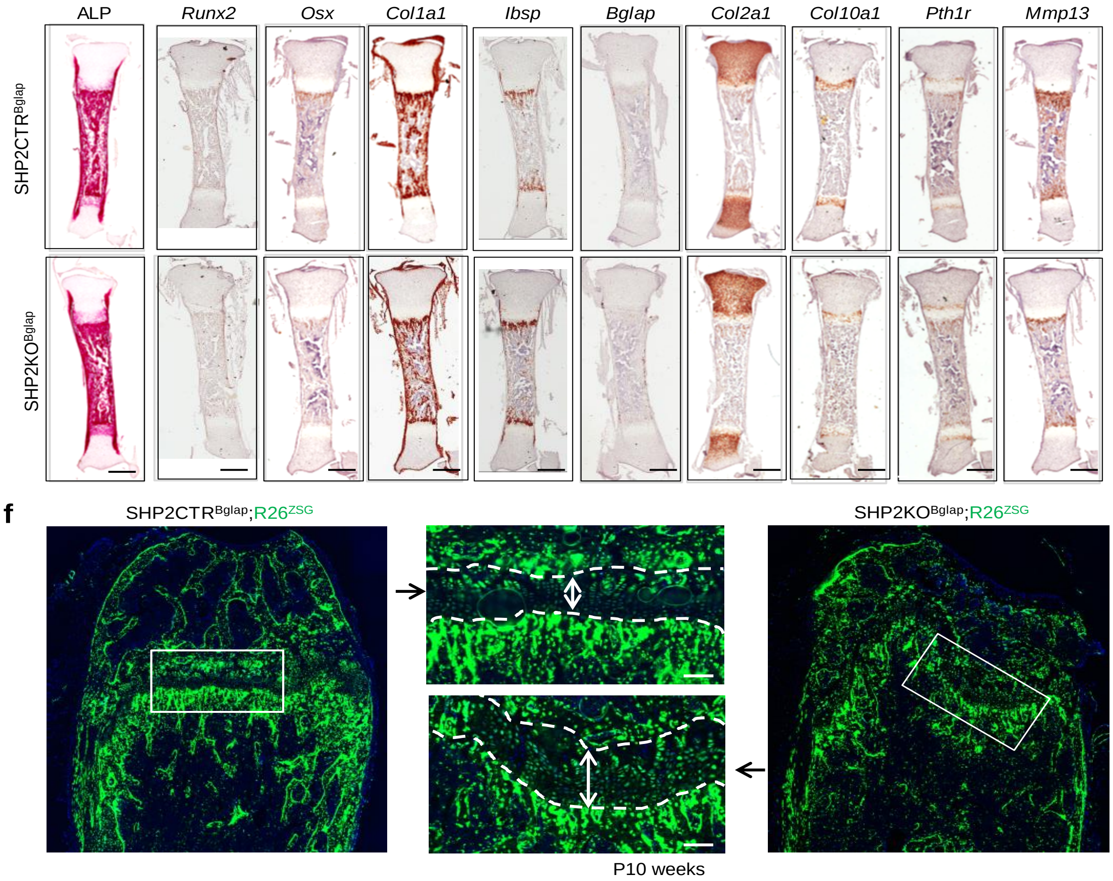

Fig. S1 Generation of mice lacking SHP2 in *Bglap*+ osteoblastic cells. a. Diagrams

depicting the breeding scheme to generate OB-specific SHP2 deficient mice and littermate controls. $R26^{ZSG}$ served as the Cre reporter when necessary. **b.** Genotypes and shorthand nomenclature for the mouse strains generated and studied. **c.** Femoral frozen sections demonstrate the distribution of *Bglap+* cells at postnatal (P) 4 weeks in mice with indicated genotypes. $SHP2CTR^{Bglap/ER}$ and $SHP2KO^{Bglap/ER}$ mice received 3 doses of TM (1mg/per mouse) injection at 2 weeks of age. Scale bar: 1mm. **d.** Images of newborn (P1.5 day) tibia frozen sections immunostained with antibodies against SHP2 (red) demonstrate the efficacy of Bglap-Cre-mediated *Ptpn11* floxed allele deletion in OBs and osteocytes. Note that SHP2 was robustly deleted in *Bglap+* OBs and osteocytes (yellow cells; arrowheads). Scale bar: 100µm. **e.** Tibia frozen sections from P1.5 day newborn mice stained for ALP activity and hybridized using the RNAscope® technology for assessing the abundance of indicated osteogenic and chondrogenic gene transcripts (n=3). Scale bars: 500µm. **f.** Lineage tracing studies demonstrate the expanded and distorted growth plate cartilage in the distal femurs of 10-week-old $SHP2KO^{Bglap}$;$R26^{ZSG}$ mice, compared to $SHP2CTR^{Bglap}$;$R26^{ZSG}$ controls. Enlarged view of the boxed areas was shown in the middle panel (n=3).

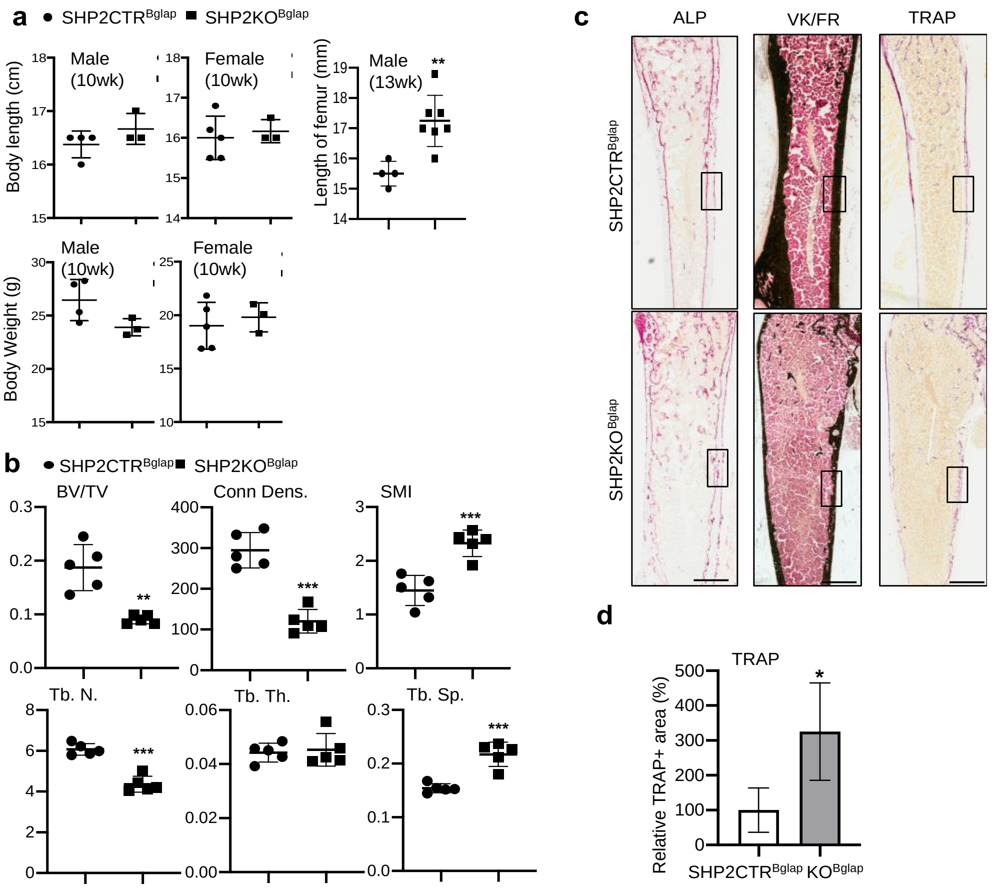

Fig. S2 SHP2 deletion in the *Bglap+* cells had no affect on body weight or length, but significantly increased tubular bone length and compromised bone volume in mice. a. Scatter plots show the distribution of body weight and length of 10-week-old male and female mice and femur length of 13-wk-old male mice with indicated genotypes. Note Both body weight and length are comparable between SHP2CTR^{Bglap} and SHP2KO^{Bglap} mice at 10 weeks of age but the femur length was increased in 13-wk-old SHP2KO^{Bglap} mice compared to SHP2CTR^{Bglap} controls. **b.** µCT-based histomorphometry results for volumetric bone density (BV/TV), connectivity density (Conn.D), trabeculae number (Tb.N), trabecular thickness (Tb.Th), trabecular spacing (Tb.Sp), and structure model index (SMI) at 10 weeks of age (n=5. **p<0.01). **c.** Representative images of ALP, Von Kossa and TRAP stained femoral sections from 13-wk-old mice. Enlarged boxed area were shown in Fig. 2b&c and Fig. 4a. Bar:1mm. **d.** TRAP-stained areas vs. the total area on the diaphyseal cortical bone (0.5 mm in length) of SHP2CTR^{Bglap} and SHP2KO^{Bglap} mice (Fig.4a) were

measured using NIH ImageJ software and presented as bar graphs (d) (%) (n=4, p<0.05; Student's t test).

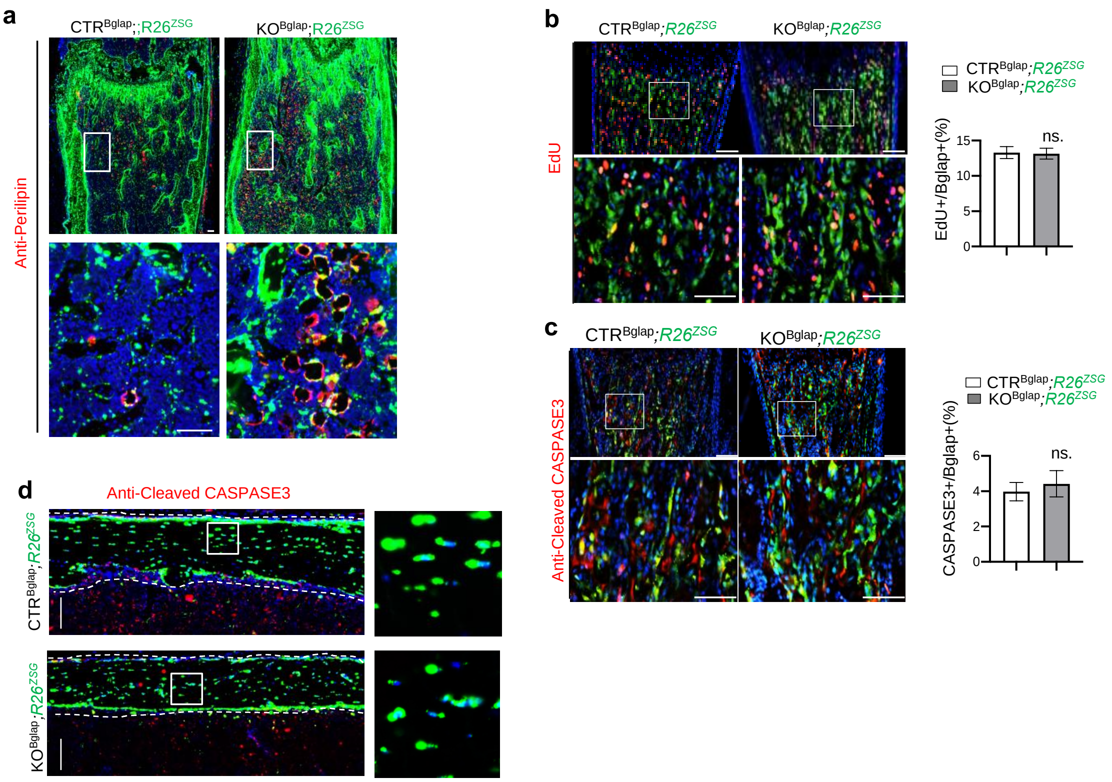

Fig. S3 SHP2 deletion in *Bglap*+ cells has no apparent effect on cell proliferation and viability. a. Femur frozen sections were immunostained with antibodies against Perilipin showing the massive accumulation of adipocytes in the bone marrow of 13-wk-old SHP2KO^{Bglap};R26^{ZSG} mice, compared to SHP2CTR^{Bglap};R26^{ZSG} controls. Enlarged view of the boxed areas shown at the bottom (n=3). Scale bar: 100μm. **b.** Tibia frozen sections demonstrating the number and distribution of EdU+ cells in the newborn tibia metaphysis determined by the EdU labeling assays. Mice received an intraperitoneal injection of EdU (20mg/kg) 2 hours before sacrifice. Enlarged view of the boxed areas shown at the bottom. Quantitative data are presented on the right (n=3; p=0.814, Student's t test.).Scale bar: 100μm. **c-d.** Tibia and femur frozen sections were immunostained with antibodies against Cleaved-CASPASE3 showing the number and distribution of apoptotic cells in the metaphysis of newborns(c) and in the cortical bone of 4-wk-old mice(d) (n=3) . Enlarged

view of the boxed areas shown at the bottom (b,,c) and on the right (d). Quantitative data are presented on the right (n=3; *p*=0.450, Student's *t* test.). Scale bar: 100μm.

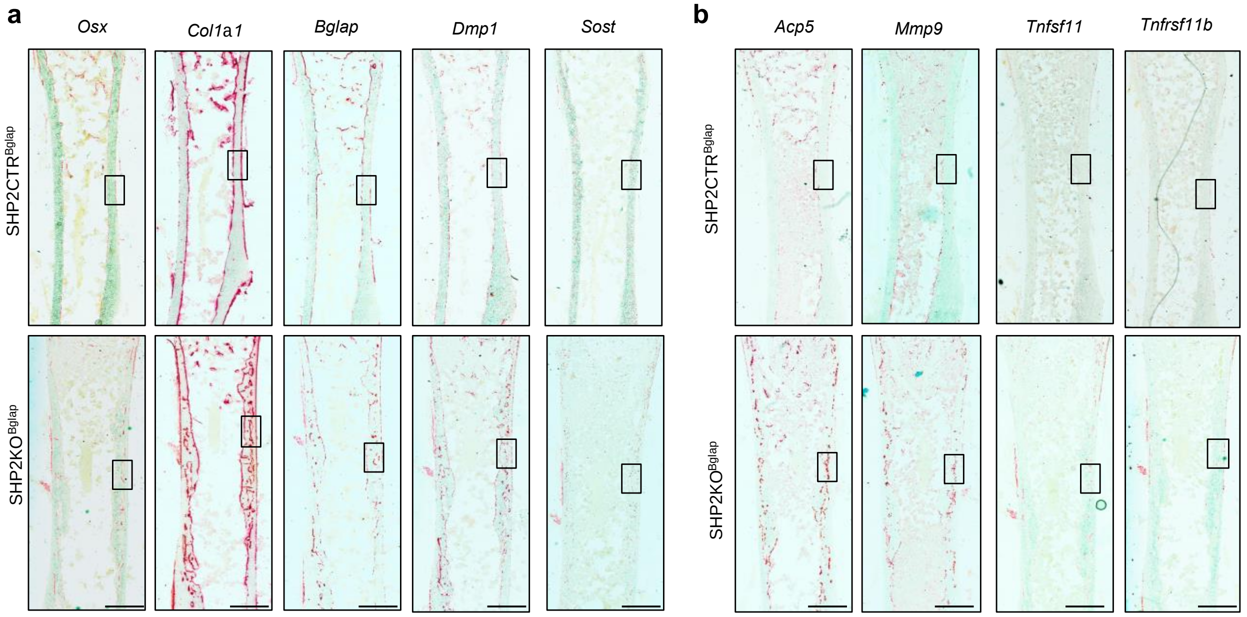

Fig. S4 RNAscope images of femoral frozen sections demonstrate the transcript abundance of indicated osteogenic (a) and osteoclastogenic (b) genes from 13-wk-old mice. Enlarged view of the boxed areas were presented in Fig. 3a and Fig. 4 b&c, respectively. Scale bars: 1mm.

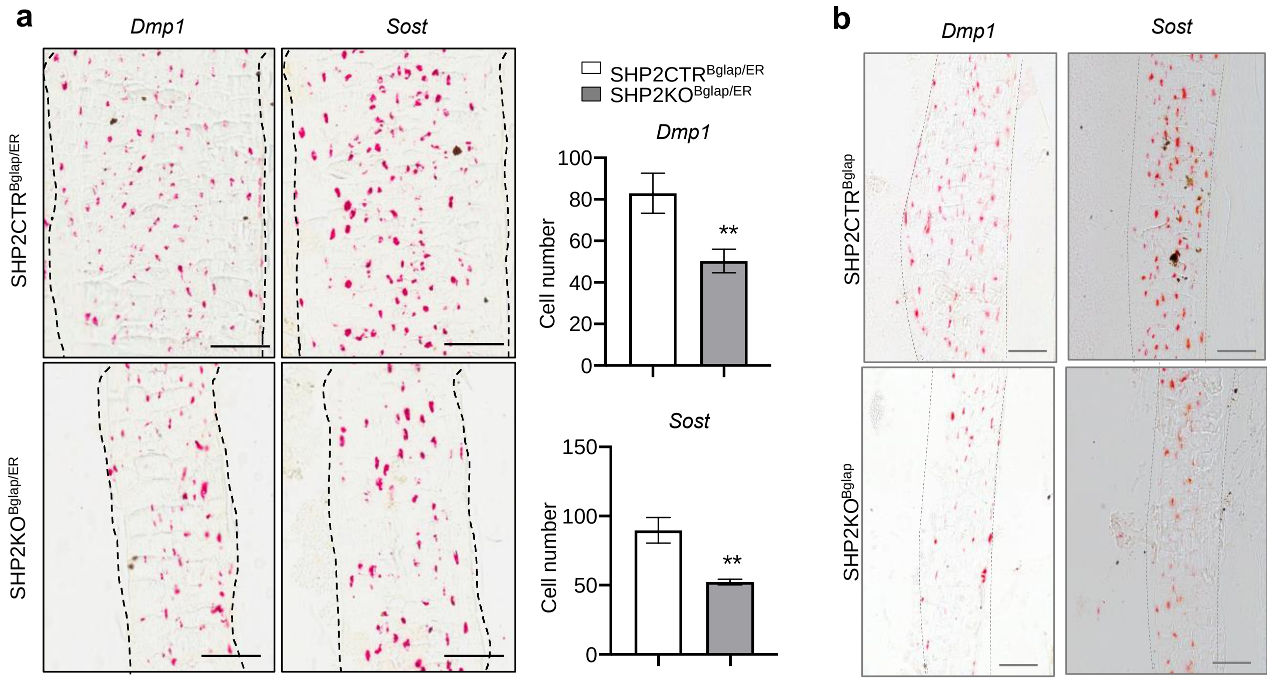

Fig. S5 SHP2 deficiency impairs osteocyte maturation. a. Femoral frozen sections show the transcript abundance of *Dmp1* and *Sost* determined using RNAScope® in 13-week-old SHP2CTR$^{Bglap/ER}$ and SHP2KO$^{Bglap/ER}$ mice that had received 3 doses of TM injection at week 2. *Dmp1* and *Sost* positive cells were counted in the diaphyseal cortical bone (500 μm x 200 μm) of control and SHP2 mutant mice. Quantified data from (a) are presented as bar graphs on the right (n=3, $^{**}p<0.01$, Student's *t*- test). **b.** Representative RNAscope images show the abundance of cells positive for *Dmp1* and *Sost* in femoral sections from 4-wk-old mice indicated.

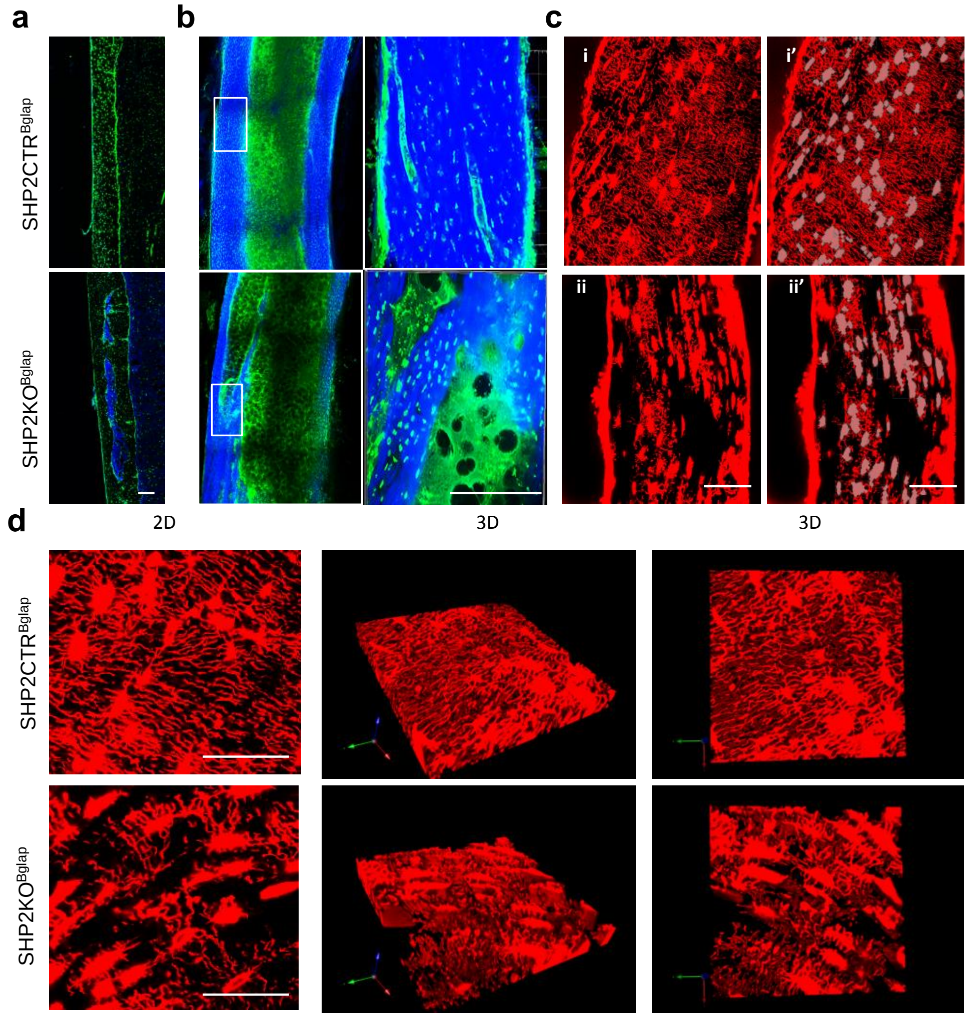

Fig. S6 SHP2 deletion impairs OB and osteocyte maturation and lacuna-canalicular system formation. a-b. Fluorescent images of femur sections from indicated 13-week-old mice demonstrate the distribution of osteocytes and unmineralized matrix in cortical bone determined using fluorescent microscope (a) and PEGASOS-based fluorescent imaging technology (b) (n=3). Enlarged views shown on the right. **c-d.** Confocal images demonstrate the morphology and distribution of osteocytes and the lacuna canalicular system (LCS, red) in the humeral cortex of SHP2CTR^{Bglap} and SHP2KO^{Bglap} mice stained by Cyanine5 NHS ester (5mg/ml for 4 hrs). The LCS in SHP2CTR^{Bglap} mice had a uniform spatial distribution and high interconnectivity in both 2D slices (c) and 3D projections (d). In contrast, the LCS in SHP2KO^{Bglap} mice was unevenly distributed and the connectivity was disrupted. 3D projections were built from thirty slices into a 11.2 μm thick tissue image.

Confocal imaging and analysis were acquired using Zeiss LSM 510 (Carl Zeiss, Thornwood, NY, USA) and the Volocity® software package (PerkinElmer, Tempe, AZ, USA), respectively. Scales: a,b: 100µm; c: 50µm; and d: 30µm.

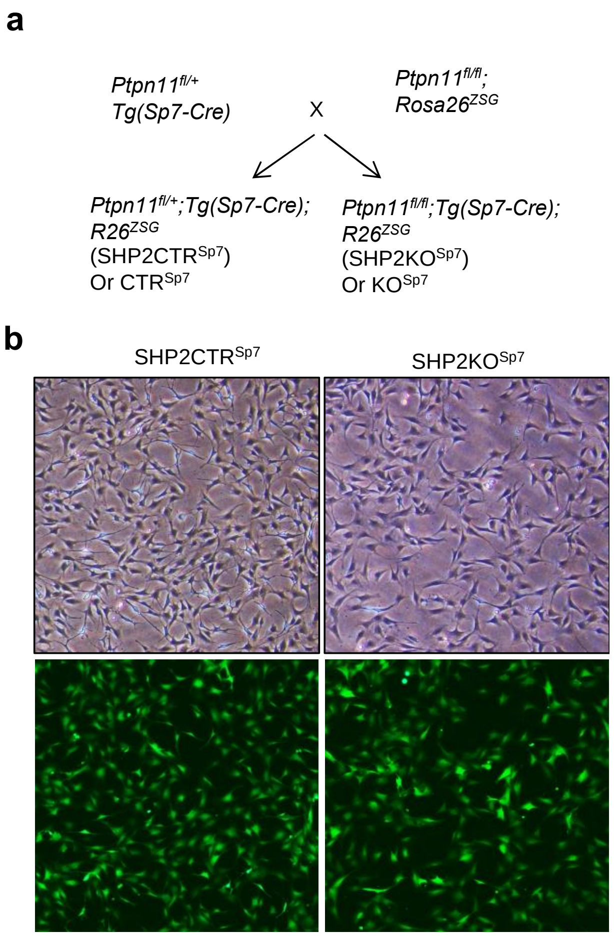

Fig. S7 Generation of control and SHP2 deficient osteoblast cell lines a. Schematic diagram denotes the breeding strategy to generate the SV40 large T antigen-immortalized osteoblasts from the SHP2CTR^{Sp7};R26^{ZSG} and SHP2KO^{Sp7};R26^{ZSG} mice. **b.** Phase-contrast and fluorescent images show the morphology of immortalized osteoblasts with the indicated genotypes.

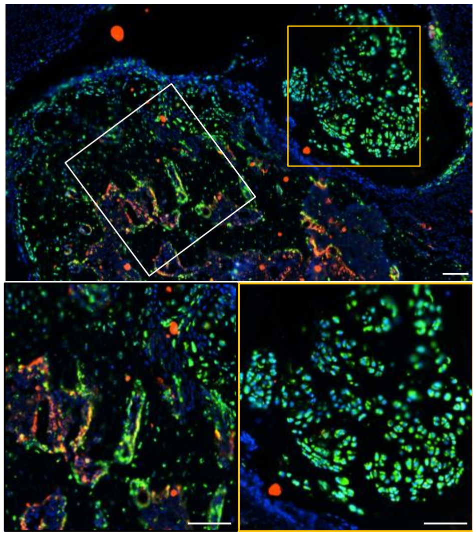

Fig. S8. Proximal femoral frozen sections from 13-week-old SHP2KO^{Bglap};R26^{ZSG} mice immunostained with SHP2 demonstrate the robust SHP2 deletion in the cartilaginous neoplastic lesions. Enlarged views of the boxed areas are shown at the bottom.

Fig. S9 A list of the primer set sequences used for qRT-PCR analysis in this study

Oscn	F	5'-TCT GAC AAA GCC TTC ATG TCC-3'
	R	5'-AAA TAG TGA TAC CGT AGA TGC G-3'
Alp	F	5'-CCC CAT AGT CAC GGC CAG TC-3'
	R	5'-TTG GGT GGG GAG TGT GTG TG-3'
RankL	F	5'-GGC CAC AGC GCT TCT CAG-3'
	R	5'-GAG TGA CTT TAT GGG AAC CCG AT-3'
Osx	F	5'-ATG GCG TCC TCT CTG CTT GA-3'
	R	5'-GAA GGG TGG GTC ATT TG-3'
Dmp1	F	5'-AGA TCC CTC TTC GAG AAC TTC GCT-3'
	R	5'-TTC TGA TGA CTC ACT GTT CGT GGG TG-3'

Fig. SV1 A video clip demonstrates the gait change in 15-week-old SHP2KO^{Bglap} mice compared to its age and sex matched SHP2CTR^{Bglap} mice. Both male and female mice display the same phenotype at this age (n=8).
